# Automatic Capture Verification in Pacemakers (Autocapture) - Utility and Problems

**Published:** 2004-04-01

**Authors:** Ruth Kam

**Affiliations:** Senior Consultant Cardiologist, National Heart Centre, Singapore

## Introduction

The concept of a closed-loop feedback system, that would automatically assess pacing threshold and self-adjust pacing output to ensure consistent myocardial capture, has many appeals. Enhancing patient safety in cases of an unexpected rise in threshold, reduced current drain, hence prolonging battery longevity and reducing the amount of physician intervention required are just some of the advantages.

Autocapture (AC) is a proprietary algorithm developed by St Jude Medical CRMD, Sylmar, CA, USA, (SJM) that was the first to commercially provide these automatic functions in a single chamber pacemaker (Microny and Regency), and subsequently in a dual chamber pacemaker (Affinity, Entity and Identity family of pacemakers).

This article reviews the conditions necessary for AC verification and performance and the problems encountered in clinical practice.

## What is Autocapture?

The AC algorithm comprises four fully automatic pacemaker functions:

1. Capture confirmation
2. Back-up high voltage pulse in case of loss of capture
3. Threshold search and documentation
4. Output regulation

### Capture Confirmation

This is achieved by the ability to detect the presence or absence of an evoked response (ER) potential by the pacemaker circuitry. There exists 2 sense amplifiers in AC pacemakers - a PR sense amplifier to sense spontaneous R waves and an ER sense amplifier to sense evoked response. After the delivery of a pacing pulse, the ER sense amplifier is blanked for 14 ms (so as to disregard the residual polarization effects of the pacing pulse) and then open from 15 to 62.5 ms in order to detect the ER. Accurate discrimination between the polarization signal (PS), which is the result of the electrical charge that remains on the lead tip after the pacing pulse has been delivered and the ER, which is produced by local myocardial capture, is crucial for its determination. A high lead polarization signal can produce so much "background noise" relative to the ER signal that the pacemaker is unable to distinguish capture from non-capture.

### Back-up pulse after loss of capture

Patient safety is enhanced by delivery of a 4.5 V/0.49 ms back-up pulse when no ER signal is detected within the 15 - 62.5 ms detection window after the pacing pulse.

### Threshold search

An automatic threshold search is initiated under one of three conditions:

- Whenever the pacemaker detects loss of capture in two consecutive beats, which it interprets as a rise in prevailing threshold.
- Every eight hours in the absence of (a).
- Manually using a programmer or a magnet.

In the Microny and Regency, the pacing output is reduced by 0.3V till two consecutive losses of capture and back-up pulses are emitted. The output is then increased by 0.3V until there are two consecutive capture confirmations. This is taken as the capture threshold.

For the dual chamber pacemakers like Affinity and Identity, during a threshold search, the AV interval is first shortened  to ensure there is no fusion with intrinsic conduction (25 ms for sensed AV and 50 ms for paced AV delay).The pacing output is reduced stepwise by 0.25 V until loss of capture and a back-up pulse is emitted. After two consecutive losses of capture at the same output, the output is increased by 0.125 volts until there are two consecutive capture confirmations at the same output. This value is then taken as the stimulation threshold. The pacemaker also documents each threshold result in the form of a graph over time.

### Output regulation

After determination of the threshold, the pacemaker adjusts its output to stimulate at 0.3V (for single chamber) and 0.25 V (for dual chamber) above the prevailing threshold. It is important to emphasise that capture confirmation is occurring on a beat-to beat basis and back-up pulse, threshold determination and output regulation then follow accordingly.

## What can impact proper operation of the AC function?

These variables may be hardware-, implant-, patient-, or programming-specific.

### Hardware- and implant-related factors

The use of a bipolar lead is mandatory for AC function. The AC pacemaker paces in unipolar mode, from tip to case,(to increase the signal to noise ratio) and senses in bipolar mode, from tip to ring, (to minimize interference from muscle activity).

Choice of a pacing electrode with low lead polarization characteristics is essential. The most important factor influencing polarization appears to be the type of coating at the lead tip rather than the tip electrode size or the type of fixation. Titanium nitride (TiN) coated electrodes have the lowest and platinum helix (PH) electrodes give the highest polarization signals [1]. As a result TiN electrodes had a higher functional rate of AC (91.7%) versus PH (0%).

Intraoperative measurement of the ER signal during lead positioning has been recommended by SJM in order to achieve the biggest ER signal relative to the PS so that at the recommended ER sensitivity setting, the PS comprises less than 60% of it. Unfortunately these measurements cannot be made with the standard pacing system analyzer (PSA). The lead has to be connected to the pulse generator and the system has to be interrogated with a sterile wand connected to the APS II or APS μ-programmer. The ER signal must be at least 4.5 mV and the polarization signal must not be more than 1.5 mV. There is also no correlation between the spontaneous R wave as measured with the standard PSA and the ER signal [2,3] . If these intraoperative measurements are not made and the final lead position is decided by the usual criteria, approximately 5 - 7% of patients were found to have inadequate ER signals and AC could not be activated [3,4].

### Patient-related factors

For AC to be activated, the pacemaker has to be able to initiate a threshold search. For single chamber pacemakers, this is done by temporarily increasing the pacing rate to 100 bpm or incrementally by 10 bpm up to a maximum of 120 bpm if the patient’s intrinsic rhythm inhibits the device. If the patient’s heart rate is above 120 bpm, e.g. atrial fibrillation with rapid ventricular response, the threshold test is postponed until the rate slows to the point when pacing is established. If the rate is persistently high, so that an automatic threshold search cannot be initiated, AC may have to be programmed OFF [4]. One solution to this would be to slow the rate with drugs. More importantly, good rate control would benefit the patient symptomatically in terms of palpitations and heart failure, besides ensuring AC function. For dual chamber pacemakers, this is usually not a problem as the AV delay is temporarily shortened during a threshold search, so as to avoid fusion beats.

Besides true loss of capture, the AC pacemaker may misinterpret certain conditions as loss of capture. This could be due to too low an ER signal (or inappropriate ER sensitivity setting relative to it), micro-dislodgement of the lead, R wave undersensing or pseudofusion beats.

 If the ER signal is too small, repositioning of the lead can easily correct the problem, provided this is known at the time of implant. If the ER signal is not routinely measured during implant, and if increasing the ER sensitivity cannot compensate for the small ER signal, then AC function cannot be enabled subsequently.

Depolarisation of the heart in fusion beats result partly from intrinsic conduction and partly from the pacing pulse, so that an evoked response may sometimes be seen. In pseudofusion beats, the heart’s intrinsic conduction system depolarizes the myocardium entirely, so that when the pacing pulse arrives, the tissue is refractory and no ER signal is detected, causing delivery of a back-up pulse. After two consecutive pseudofusion beats, a threshold search is initiated. When capture is confirmed, the output is then increased to 0.25 V above the threshold. However if the output climbs to more than 4.2 volts, the pacemaker will stimulate at high output mode (HOM) or 4.5 V at 0.49 ms. It will then stay there until the next time a threshold search is initiated. The presence of frequent pseudofusion beats will result in many threshold searches being initiated. Some will give normal threshold values if pseudofusion beats were not present during the search and some will end up in HOM if capture losses were due to pseudofusion beats. This may not necessarily lead to excessive energy wastage if the pacemaker is predominantly inhibited, but it can cause confusion during follow-up especially if there are frequent oscillations of threshold due to pseudofusion beats.

A fusion avoidance algorithm, which is inherent in the AC pacemakers, resets the timing circuit from the back-up pulse to slow the pacing rate and allow intrinsic beats to come through. For dual chamber versions, this fusion avoidance algorithm consists of extending the AV delay in the next beat following a back-up pulse, by as much as 100 ms. An understanding of these algorithms is essential when interpreting Holter recordings of patients with these types of pacemakers, as these are often misinterpreted as pacemaker malfunction.

### Programming related factors

R wave undersensing, similarly, may cause the pacemaker to stimulate in the refractory period of the ventricle and  fail to capture, thus leading to many threshold searches and possibly HOM. This problem can be easily corrected by increasing the PR sensitivity appropriately.

In the case of pseudofusion beats causing mistaken "loss of capture", programming a hysteresis rate at least 10 beats per minute below the lower rate will prevent competition with the patient’s intrinsic rhythm and the presence of pseudofusion beats. In the case of a dual chamber pacemaker with intrinsically conducted beats, the "Autointrinsic Conduction Search" option can be turned on to automatically prolong the AV interval when the pacemaker detects an intrinsically conducted beat and thus avoid pseudofusion.

However, interactions between these algorithms and the inherent fusion avoidance algorithms occasionally lead to complex pacing behaviour that make ECG interpretation difficult for the clinician [5].

## Clinical Experience

Extensive clinical experience has been obtained with the AC algorithm in several multicentre studies. In both the European [2] as well as the North American [6] studies, which followed patients up to one year, the algorithm was found to be safe and effective and did not result in exit block or other adverse events. If anything, the algorithm tended to err on the side of safety, providing back-up pulses in all cases where there was presumed loss of capture.

A case report appeared in 2001, concerning a patient, in whom failure of emission of back-up pulse after loss of capture, resulted in 8 seconds of ventricular standstill [7]. This was later discovered to be due to a transient threshold rise in the patient, which coincided with an automatic function in the pacemaker, which measured battery current drain every 11.25 minutes for 10 seconds, during which time the back-up pulse was not available. This was finally rectified in that patient, using a software correction to reduce the frequency of that measurement to once every 24 hours and provide an automatic increase in output by 1V during the measurement. While this may be an isolated report in the literature, it emphasizes the need for close post-marketing surveillance of any new technology or features in any pacemaker or defibrillator, before we embrace it fully or relax on our previous follow-up practices.

## Future Directions

### Use with other leads without low polarization characteristics

Modification of the pacing pulse has been shown in one experimental study to improve the PS, such that pacing leads without low polarization characteristics could have a better ER to PS ratio and hence, AC function could be enabled in a higher proportion of these leads [8]. The pacing pulse conventionally used is a biphasic pulse with an initial negative deflection, followed by a rapid discharge in the positive direction to counteract the effect of the polarization charge. Increasing the duration of the fast positive second phase from 6 ms to 10ms significantly reduced the polarization voltage in all leads but especially in leads with high polarization characteristics. This has favourable implications for pacemaker replacements so that AC function can be used with pre-existing leads from other manufacturers.

### Use with epicardial leads

In paediatric patients, epicardial pacing electrodes are frequently necessary because of their small size and the need to preserve their veins for future use when they reach a suitable size for transvenous implants. The higher pacing threshold, when compared to endocardial leads, is of concern, because of higher current drain and hence more frequent battery replacements. The use of steroid-eluting bipolar epicardial leads from Medtronic with the Regency SR+ pacemaker was evaluated and found to be feasible in 12 of 14 children (86%)s [9,10].

### Use with implantable defibrillators

An acute study [11], looking at the feasibility of using the AC algorithm in a group of patients undergoing ICD implantation, reported that evoked response and polarization signal amplitudes using standard, single coil, true bipolar ICD leads were sufficient to allow the AC algorithm to function in 20 of 21 patients (95.2%). However, with dual coil ICD leads and integrated bipolar sensing, AC could only be functional in 2 out of 9 patients (22.2%). Unfortunately, since the present AC algorithm requires unipolar pacing, this is not recommended for use with ICD systems in case it interferes with sensing of ventricular fibrillation.

### Other threshold tracking algorithms

Although autocapture is the first algorithm to provide automatic threshold tracking and output regulation, other pacemaker manufacturers have also come up with competing algorithms. These are Capture Control in Biotronik, Ventricular Capture Management in Medtronic and Automatic Capture in Guidant pacemakers. There are important differences between these algorithms.

With Biotronik, ER detection also requires a bipolar lead with low polarization characteristics. There is no back-up safety pulse during loss of capture and output is increased in 2V steps if there is persistent loss of capture. After a programmable length of time, the output is decreased to its original value to test if capture can be obtained with a lower output. This kind of algorithm ensures safety in unexpected threshold rise but will not improve battery longevity.

Other manufacturers (Medtronic and Guidant) have devised alternative methods of capture verification, which can operate with both unipolar and bipolar leads without low polarization characteristics. These rely on modifications to the sense amplifiers to discriminate between the ER and PS, but clinical experience with these algorithms is far less extensive and has not been systematically reported to the same extent as the original AC algorithm.

## Atrial autocapture

Currently, the AC algorithm is only available in the ventricle, but there is considerable interest in applying it to the atrium as well. There are potentially more difficulties with AC verification in the atrium because the atrial evoked response is considerably smaller than the ventricle and differentiation between the PS and ER signal is significantly more problematic. Early attempts were made by Curtis [12], using a triphasic pulse to minimize polarization signals. However the pacing circuitry itself would consume so much energy as to negate any potential savings from reduction of the pacing output. One practical approach may be to use the atrial evoked response integral ("area under the curve") and using bipolar sensing (tip to ring) and unipolar pacing [13]. Another approach, using different pairs of electrodes for pacing and sensing (e.g. unipolar pacing from atrial tip electrode and bipolar sensing from the atrial ring electrode to the can or an indifferent electrode) has been described [14] but the disadvantage is that detection of the atrial ER may be so delayed,due to the time needed for the impulse to propagate to the sensing electrode, as to preclude timely delivery of a backup pulse in the event of loss of capture.

## Conclusion

Autocapture has been shown to be safe and effective. However, to reap the full potential of all its benefits, one must understand its limitations and pitfalls and the ways to overcome these. "To implant and forget" is a dream not to be realized, even with this advanced technology.
